# Laser microdissection-based analysis of the Y sex chromosome of the Antarctic fish *Chionodraco hamatus* (Notothenioidei, Channichthyidae)

**DOI:** 10.3897/CompCytogen.v9i1.8731

**Published:** 2015-02-05

**Authors:** Ennio Cocca, Agnese Petraccioli, Maria Alessandra Morescalchi, Gaetano Odierna, Teresa Capriglione

**Affiliations:** 1 Istituto di Bioscienze e Biorisorse, CNR, via P. Castellino 111, 80131 Napoli, Italy; 2 Dipartimento di Biologia, Università di Napoli Federico II, Complesso Universitario Monte S. Angelo, via Cinthia, 80126 Napoli, Italy

**Keywords:** Sex chromosomes, Antarctic fish, CHD1 gene

## Abstract

Microdissection, DOP-PCR amplification and microcloning were used to study the large Y chromosome of *Chionodraco hamatus*, an Antarctic fish belonging to the Notothenioidei, the dominant component of the Southern Ocean fauna. The species has evolved a multiple sex chromosome system with digametic males showing an X1YX2 karyotype and females an X1X1X2X2 karyotype. Fluorescence in situ hybridization, performed with a painting probe made from microdissected Y chromosomes, allowed a deeper insight on the chromosomal rearrangement, which underpinned the fusion event that generated the Y. Then, we used a DNA library established by microdissection and microcloning of the whole Y chromosome of *Chionodraco hamatus* for searching sex-linked sequences. One clone provided preliminary information on the presence on the Y chromosome of the CHD1 gene homologue, which is sex-linked in birds but in no other vertebrates. Several clones from the Y-chromosome mini-library contained microsatellites and transposable elements, one of which mapped to the q arm putative fusion region of the Y chromosome. The findings confirm that interspersed repetitive sequences might have fostered chromosome rearrangements and the emergence of the Y chromosome in *Chionodraco hamatus*. Detection of the CHD1 gene in the Y sex-determining region could be a classical example of convergent evolution in action.

## Introduction

The chromosome microdissection and microcloning technique (Almeida-Toledo et al. 2000) can be used for a number of applications, including generation of chromosome painting probes and construction of genetic linkage as well as physical maps. First applied to *Drosophila melanogaster* (Meigen, 1830) polytene chromosomes and later employed to obtain whole or partial human chromosome paints (Jauch et al. 1992, Stanyon et al. 1995, Bigoni et al. 1997, Morescalchi et al. 1997, Yang et al. 2006), chromosome microdissection is considered a suitable bridge between cytogenetics and molecular genetics (Zhou and Hu 2007). Chromosome microdissection has recently been applied to lower vertebrates to generate painting probes for phylogenetic studies (Diniz et al. 2008, Giovannotti et al. 2009, Machado et al. 2011, Pokorná et al. 2011, Trifonov et al. 2011, Kawagoshi et al. 2012). In cold-blooded vertebrates its application may be hampered by difficulties in identifying the chromosomes clearly in the karyotype of the species, where the heteromorphic sex chromosome is often the one most easily recognizable (Almeida-Toledo et al. 2000, Odierna et al. 2004, Kuprijanova et al. 2005, Cioffi et al. 2012). Sex chromosomes have originated independently many times in both animals and plants from ordinary autosomes (Arnemann et al. 1986, Almeida-Toledo et al. 2000). Their evolution is characterized by a loss of gene function on the non-recombining Y chromosome, as seen in many taxa (Badenhorst et al. 2012, Böhne et al. 2014).

While sex-determining genes have been identified and mapped on the heteromorphic sex chromosomes of mammals and birds (Handley et al. 2004, Lahn and Page 2004), data on their linkage and localization in cold-blooded vertebrates are only just beginning to be reported.

Fish sex chromosomes often appear undifferentiated, sharing a similar morphology and a comparable gene composition (Charlesworth 2004, Charlesworth et al. 2005). The sex-determining region (SDR) has been identified in several teleost species (Geschwend et al. 2012, Liew et al. 2012) and the first likely sex-determining gene was found in medaka, *Oryzias latipes* (Temminck & Schlegel, 1846), in which a copy of the Dmrt1 gene, DmY, maps on the putative Y sex chromosome. Some correspondence has been found, in other fish as well as for the congeneric species (Matsuda 2005, Kondo et al. 2009), while novel candidates, all coding for members of the TGF-beta signalling pathway, have recently been identified in different teleostean species (Kikuchi and Hamaguchi 2013, Bohne et al. 2014).

Some fishes present multiple sex-chromosome systems, in which the initial stage of differentiation seems to be independent of heterochromatin accumulation in chromosomes and to be related to chromosome rearrangement, whereas simple sex-chromosome systems show progressive heterochromatin accumulation (Almeida-Toledo et al. 2000, Cioffi et al. 2012).

The variety of sex-chromosome systems and the broad range of sex-determination mechanisms typical of fishes make it an interesting group in which to explore sex-chromosome evolution. The present study was designed to seek correlations between cytologically recognizable fish X and Y chromosomes and to establish whether they are related to different genetic make-up or simply to fusion events, perhaps trigged by differential amplification of repetitive DNA fractions.

To shed light on the genome composition of a teleost sex chromosome, laser microdissection and pressure catapulting (LMPC) was used to isolate the Y chromosome from standard mitotic plates of the Antarctic icefish *Chionodraco hamatus* (Lönnberg, 1905) belonging to the Notothenioidei, the dominant endemic component of the Southern Ocean fauna. The physiological and molecular adaptations that have enabled their evolution have made this Antarctic clade unique among vertebrates. The karyotype of most Notothenioidei shows 2n = 48 mostly acrocentric chromosomes, which is considered basic for Perciformes, and nucleolus organizer regions (NORs) localized on the telomeres of a single chromosome pair (Morescalchi et al. 1996, Pisano and Ghigliotti 2009). Heteromorphic sex chromosomes have been found in species belonging to three notothenioid families (Morescalchi et al. 1992, Caputo et al. 2002). In such species males have a large Y chromosome, resulting from a centric or tandem fusion event, and a consequent decrease in chromosome number to 2n = 47. This feature is shared by *Chionodraco hamatus*, the model selected for this study. *Chionodraco hamatus* females have 2n=48 chromosomes with a small metacentric pair, a submetacentric pair, 20 acrocentric pairs and a X_1_X_1_X_2_X_2_, multiple sex chromosome system, where X_1_ is submetacentric and X_2_ is acrocentric; males of *Chionodraco hamatus* present 2n=47 with the same basal chromosome set plus the X1X2Y sex chromosome system, where the Y is the largest submetacentric element, likely, derived from X1-X2 tandem fusion (Morescalchi et al. 1992).

*Chionodraco hamatus* Y chromosomes were isolated and amplified by degenerate oligonucleotide primed PCR (DOP-PCR) as described by Telenius et al. (1992). The DNA fragments generated were (i) labelled with biotin, hybridized *in situ* to metaphase spreads, and (ii) used to construct a Y-chromosome mini-library that was subsequently screened for Y-chromosome DNA sequences.

## Methods

### Y chromosome microdissection

*Chionodraco hamatus* specimens were collected near Mario Zucchelli Station, on the coast of Terra Nova Bay (74°42'S, 164°07'E), during Italian Antarctic expeditions in 2000 and 2005. They were killed by severing the spinal cord according to animal welfare guidelines.

Mitotic chromosome plates were prepared from a *Chionodraco hamatus* male as described in Morescalchi et al. (1992). The chromosome suspension was dropped onto slides covered with a polyethylene naphthalate membrane, and Y chromosomes were microdissected using a PALM MicroBeam system (Carl Zeiss, Italy, http://www.zeiss.it/). All procedures were as described by Schermelleh et al. 1999.

Y chromosomes (20/slide) were outlined and excised using a low-energy laser beam and catapulted by a single laser pulse directly into the cap of an Eppendorf tube, to minimize contamination.

### DOP-PCR

DOP-PCR reactions were carried out without chromosome pre-treatment (Telenius et al. 1992). The DOP primer sequence 5’-CCGACTCGAGNNNNNNATGTGG-3' was used to conduct a PCR reaction with 1 × ThermoSequenase reaction buffer, 40 μM dNTPs, 2 μM DOP primer, and 10 U ThermoSequenase enzyme.

The first amplification was carried out by RAMP-PCR: 94 °C for 5 min; 12 cycles at low stringency (94 °C for 90 s, 32 °C for 2 min, increasing by 0.2 °C/s to 72 °C, then 72 °C for 2 min), followed by 35 cycles at high stringency (94 °C for 90 s, 52 °C for 90 s and 72 °C for 90 s). 10 μl of the DOP-PCR product was run on 1% agarose gel.

### Fluorescence *In Situ* Hybridization (FISH)

#### FISH with the Y probe

The species specificity of the probe was checked by dot-blot hybridization under stringent conditions using digoxigenin-labelled Y chromosome material as a probe on human and fish total genomic DNA spotted on a nitrocellulose membrane. Dot-blot analysis confirmed the absence of contamination, showing a strong hybridization signal only to *Chionodraco hamatus* genomic DNA (data not shown).

The DOP-PCR-amplified chromosome material was re-amplified and labelled with 16-dUTP-biotin (Roche). The PCR mix comprised 1 x *Taq* polymerase buffer (2 mM MgCl_2_), 40 μM dATP, dGTP and dCTP, 28 μM dTTP, 12 μM 16-dUTP biotin, 2 μM DOP primer, and 0.05 U/μl *Taq* polymerase under the following conditions: (1×) 94 °C for 5 min; (35×) 90 °C for 1 min and 30 s, 52 °C for 1 min and 30 s, 72 °C for 1 min and 30 s, and (1×) 72 °C for 5 min. The FISH procedure was performed under high stringency conditions (50% formamide, 2 × SSC, 10% dextran sulphate). Metaphase spreads for FISH were prepared on ethanol-cleaned slides, air-dried and incubated overnight at 37 °C. Chromosomes were denatured in 70% deionized formamide 2 × SSC at 72 °C for 7 min and dehydrated in an ethanol series.

A DNA mixture of approximately 400 ng of the chromosome painting probe and 10 μg of the Cot-1 fraction of *Chionodraco hamatus* (Yan et al. 2010) was prepared, ethanol precipitated, vacuum dried, and dissolved in 10 μl of hybridization mixture containing 50% deionized formamide, 10% dextran sulphate, and 2 × SSC. After denaturation (10 min at 75 °C) the probe was dropped onto a slide and spread over the hybridization area using a 22 × 22 mm glass coverslip. The edges of the coverslip were sealed with rubber cement and slides were incubated for 3 days at 37 °C in a humid chamber.

Post-hybridization washes were in 0.4 × SSC/0.1% Triton X-100 for 5 min at 42 °C and in 2 × SSC/0.1% Triton X-100 for 15 min at room temperature. Signal detection was accomplished using AlexaFluor-Streptavidin 488 (Molecular Probes) according to Cocca et al. (2011). Chromosomes were counterstained with propidium iodide in Vectashield mounting medium (Vector), analysed using a fluorescence microscope (Zeiss Axioskop) equipped with a CCD camera (Hamamatsu Chilled), and processed with the Isis FISH imaging system (v. 3.04, Metasystems).

#### Mini-library construction

DOP-PCR fragments were purified with the NucleoSpin Extract II Kit (Macherey-Nagel). Three μl aliquots were cloned into the TOPO TA Cloning Kit (Invitrogen Life Technologies). Transformation and clone analysis were conducted according to the manufacturer’s instructions. Recombinant clones were isolated and PCR amplification was used to establish insert presence and size. Sequencing was performed by Primm Biotech (Milano, Italy). The nucleotide sequences, ranging from 200 to 800 bp, were used for homology searches in four databases (National Center for Biotechnology Information, NCBI: http://www.ncbi.nlm.nih.gov; DNA DataBank of Japan, DDBJ: http://www.ddbj.nig.ac.jp; Nucleotide EST Database; and RepBase: http://www.girinst.org/repbase) using BLAST (Basic Local Alignment Search Tool, http://blast.ncbi.nlm.nih.gov), FASTA (http://www.ebi.ac.uk/Tools/sss/fasta), and RepeatMasker (http://www.repeatmasker.org) sequence alignment programs.

## Results

**Figure 1.**
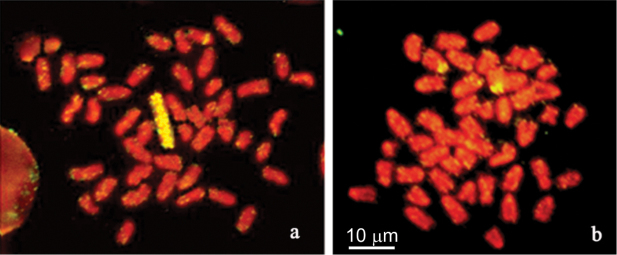
Mitotic chromosomes of *Chionodraco hamatus*. FISH with the Y chromosome paint on *Chionodraco hamatus* **a** male (2n = 47 and X1X2Y sex-chromosome system) and **b** female (2n = 48) metaphase spreads.

The results of FISH, performed on metaphase plates of *Chionodraco hamatus* males and females using the newly generated Y-chromosome paint, are shown in Fig. 1. In males (Fig. 1a) a strong hybridization signal was detected along the Y chromosome. On other chromosomes the signal was faint up to the subtelomeric regions. A single medium-sized acrocentric chromosome showed a clear pericentromeric signal. On female metaphase plates (Fig. 1b), the hybridization signal was intense and confined to the pericentromeric and interstitial regions of two medium-sized acrocentrics. Thirty sequences were recovered by cloning with clear information obtained only for four of them.

The identification of syntenic regions may have been hampered by sequence length and by the lack of whole sequenced genomes of Antarctic fishes. Most clones were found to contain redundant repetitive sequences; moreover, several clones did not show significant homologies to any genomic or translated sequence contained in the GenBank database, suggesting that they may correspond to untranslated or non-conserved regions of genes already known from other species.

**Figure 2.**
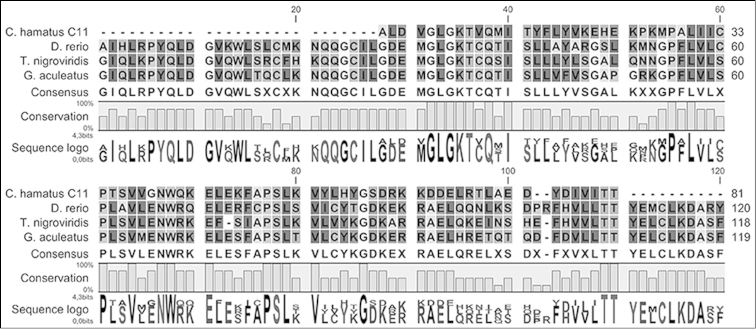
Alignment of the N-terminal portion of a Chromodomain-helicase-DNA-binding protein 1-like (CHD1) with the homologous CHD1 sequences of fish: *Danio rerio*, *Tetraodon nigroviridis*, and *Gasterosteus aculeatus*.

FASTX analysis of clone C11 (251 bp) against library ‘B170.0 vertebrates’ yielded the highest similarity score for fish genome fragments. Interestingly, its uninterrupted amino acid translation was found to be homologous to an N-terminal portion of a Chromodomain-helicase-DNA-binding protein 1-like (CHD1) (The European nucleotide archive: at http://www.ebi.ac.uk/ena/data/view/LM999957), a conserved protein with a putative role in chromatin architecture (Fig. 2).

**Figure 3.**
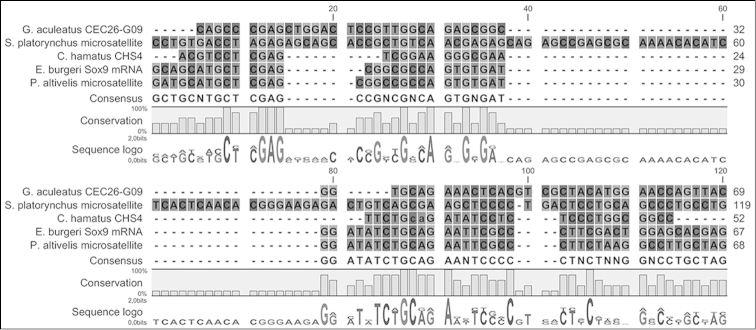
Repetitive sequence of clone CHS4 aligned with several loci of *Plecoglossus altivelis*, *Gasterosteus aculeatus*, *Scaphirhynchus platorynchus* and *Eptatretus burgeri*. Notably, in *Eptatretus burgeri* the sequence is found in Sox9.

RepeatMasker analysis allowed identification in clone CHS4 of a microsatellite strand whose sequence is conserved in other teleost species. FASTA and BLAST analysis showed that our sequence aligned with several loci in the genome of *Plecoglossus altivelis* (Temminck & Schlegel, 1846), *Gasterosteus aculeatus* (Linnaeus, 1758), *Scaphirhynchus platorynchus* (Rafinesque, 1820), and *Eptatretus burgeri* (Girard, 1855). Notably, in *Eptatretus burgeri* the sequence is found in Sox9 mRNA for Sry-box protein 9 (Ota et al. 2007) (Fig. 3). Moreover, a similar sequence is found in the ATP-binding cassette of *Trematomus newnesi* (Boulenger, 1902), another notothenioid species.

In two other clones, C3 (825 bp) and C5 (244 bp), putative ancient LINE (Long Interspersed Element) fragments (non-LTR retrotransposon of the L1/CIN4 family) were found.

**Figure 4.**
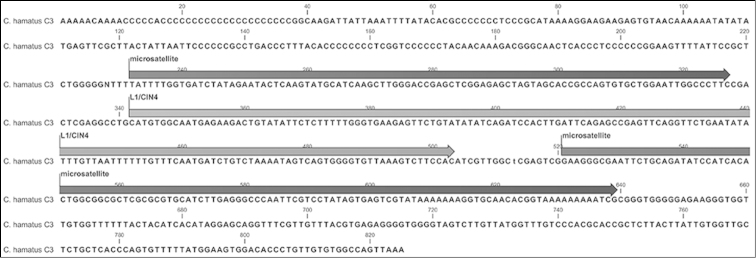
Structure of clone C3: the partial LINE (position 341–504 bp) is bordered by microsatellite regions both upstream (232–325 bp) and downstream (520–640 bp).

**Figure 5.**
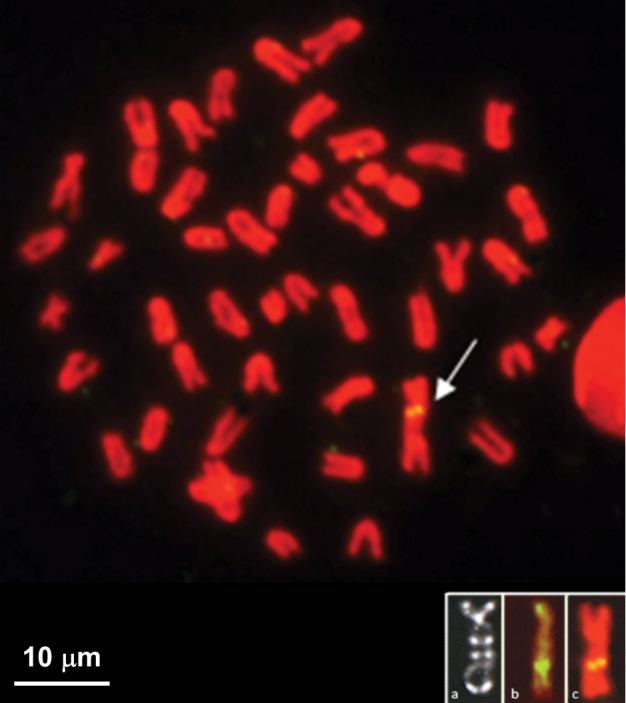
*In situ* hybridization of the C3 probe (CIN1/L4) to the *Chionodraco hamatus* male karyotype: the probe clearly maps interstitially on the q arm of the Y chromosome. Insert: **a** C-banded and chromomycin-stained Y chromosome of *Chionodraco hamatus* and **b** FISH performed using the Tc1-like DNA transposon as probe (modified from fig. 5 of Capriglione et al. 2002) compared to **c** C3 hybridized Y chromosome.

In clone C3 the partial LINE (position 342–503 bp) is bordered by repetitive regions both upstream (232–327 bp) and downstream (521–639 bp) (Fig. 4). FISH disclosed that the clone clearly mapped interstitially on the q arm of the Y chromosome (Fig. 5).

## Discussion

Chromosome microdissection provides valuable chromosome information on lower vertebrate, fish, amphibian, and reptilian genomes, in which chromosome identification is hampered by difficulties in obtaining specific chromosome markers, and detection of syntenic regions is prevented by the lack of sequenced genomes.

*Chionodraco hamatus* Y-chromosome paint hybridized strongly to a single acrocentric chromosome pair, perhaps X2X2, in the female karyotype. The signal localization on the pericentromeric region suggests that, at least in this species, sequences, which are still found on the Y chromosome, might have been accumulated on the female X_2_X_2_ pair after sex chromosome emergence.

Because the heteromorphic Y is probably the result of a tandem fusion of autosomes and morphologically undifferentiated sex chromosomes, this event may have been followed by a reduction in recombination and by formation of new linkage groups between genes that were originally found on different chromosomes, including sexually antagonistic ones (Cioffi et al. 2012, Yano et al. 2014).

The interstitial chromomycin-positive heterochromatic region of the q arm, corresponding to the region involved in the tandem fusion event (Morescalchi et al. 1992), seems to be a good candidate for the breakpoint of rearrangement, also due to the presence of a selective, preferential accumulation of repetitive sequences and transposable elements.

In a previous paper we mapped a mobile element belonging to the Tc1/mariner DNA transposon family on the q arm of the Y chromosome (Capriglione et al. 2002). The L1/CIN4 LINE isolated from the present Y-specific mini-library is also located interstitially on the long arm of the Y chromosome and might be part of this segment. These multiple ancient L1 lineages, which are occasionally found in the majority of teleost genomes, might represent the ancestral state of vertebrate L1 and are often still active (Furano et al. 2004). These elements are known to provide the machinery for retro-duplication, promoting the trafficking of particular genes on and off the sex chromosomes (Geschwend et al. 2012). Their location suggested that these mobile elements and, perhaps, other as yet unidentified elements, may have constituted a hotspot for chromosome rearrangement and Y shaping, playing a role in the induction of chromosome rearrangement and the appearance of the Y chromosome in *Chionodraco hamatus*. This Y-specific repetitive sequence could also be involved in Y chromosome differentiation in *Chionodraco hamatus*, since differential accumulation of repeats, i.e. microsatellites, is one of the earliest stages of sex-chromosome differentiation (Jones and Singh 1985, Arnemann et al. 1986, Schlötterer 2000, Kubat et al. 2008, Pokornà et al. 2011).

Repetitive sequences preserved in teleost species have helped to identify conserved genetic synteny and measure the degree and patterns of sex-chromosome differentiation in comparative genomic studies of fish (Nanda et al. 1990, Brunelli et al. 2008, Cioffi et al. 2011, Shikano et al. 2011).

The microsatellite sequence isolated from the *Chionodraco hamatus* Y chromosome in our study appears to be conserved. In addition, it aligned with several loci in the genome of ancient and modern fishes, including *Trematomus newnesi*, an Antarctic notothenioid.

The presence of Y chromosome-specific microsatellites is well established in literature, mainly in mammals and the variability of their loci has been widely exploited to generate haplotypes for phylogenetic and forensic analysis (Goldstein et al. 1995, Sun et al. 2009). In lower vertebrates this correlation is less clear, even though the accumulation of Bkm repeats containing tandem arrays of GATA tetranucleotides on the degenerated W chromosomes of advanced snakes is one of the most extensively studied events of sex chromosome differentiation.

In fish, comparative genomic studies have shown that some of these microsatellite sequences, preserved in different teleost species, can help to examine conserved genetic sinteny, to clarify phylogeny as well as to measure the degree and patterns of sex chromosome differentiation (Brunelli et al. 2008, Cioffi et al. 2011, Yano et al. 2014).

Finally, although the present data do not allow any hypothesis to be advanced, it is intriguing that in *Eptatretus burgeri* this sequence is found in Sox9 mRNA for Sry-box protein 9, which together with steroidogenic factor 1 regulates transcription of the anti-Muellerian hormone (AMH) gene (Ota et al. 2007).

With regard to the partial *CHD1* gene sequences found in one of our Y clones, two *CHD1* gene homologues have been described in birds, which are characterized by ZW female heterogamety. In birds these homologues are associated with the heteromorphic sex-chromosome pair, one being W-linked and the other Z-linked (Fridolfsson and Hellegren 2000). However, *CHD1* genes have not been found to be sex-linked in eutherian mammals or in other organisms.

Even though the SDR has been identified in some teleost species (Froschauer et al. 2002, Volff and Schartl 2002, Volff et al. 2003), little is known about the loss and gain of fish genes involved in the molecular dynamics of the sex-specific region of sex chromosomes. The mechanisms controlling sex determination and differentiation in lower vertebrates remain unclear, since different genes seem to be involved, depending on species. For this reason it is difficult to say whether the partial *CHD1* homologue isolated in the *Chionodraco hamatus* Y mini-library might belong to the Y chromosome regions linked to sex differentiation. Further sequencing studies are needed to establish this.

## Conclusions

The data seem to confirm that sex chromosomes arose independently in distant taxa several times during fish radiation (Matsubara et al. 2006, Charlesworth and Mank 2010). In *Chionodraco hamatus*, interspersed repetitive sequences and transposable elements seem to have played a pivotal role in promoting the first steps of chromosome rearrangement and the origin of the Y chromosome. In contrast, the present data do not allow ascribing the presence of the *CHD1* gene, related to the sex chromosomes only in avian genomes, to the Y-chromosome SDR. Of course, this finding may merely be the result of convergent evolution. The repetitive sequences isolated in this study may be a starting point to sequence the entire male-specific region of *Chionodraco hamatus*, and further research may provide additional data on Y-chromosome gene linkage and genome organization.
